# Supplementary material for: CTX-CNF1 Recombinant Protein Selectively Targets Glioma Cells In Vivo
Source: Toxins (Basel). 2021 Mar 8;13(3):194. doi: 10.3390/toxins13030194 (PMC7998600; doi:10.3390/toxins13030194)
Supplement: Supplementary file 1 [file toxins-13-00194-s001.pdf]

Supplementary Materials: CTX-CNF1 Recombinant Protein Selectively Targets Glioma Cells In Vivo

Eleonora Vannini, Elisabetta Mori, Elena Tantillo, Gudula Schmidt, Matteo Caleo and Mario Costa

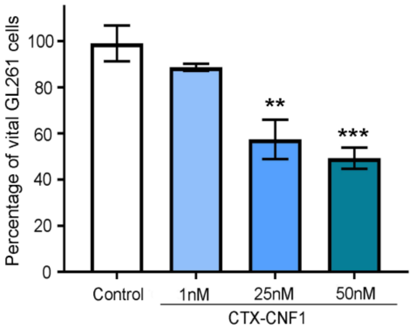

Figure S1. CNF1 affects GL261 cells vitality as reported from previous studies. Control (white, PBS) and 48 h after CNF1 treatment of at three different concentrations (i.e., 1 nM, 25 nM, 50 nM). Data represent means ± SEM, One Way ANOVA $p < 0.001$. **, $p < 0.01$; ***, $p < 0.001$.

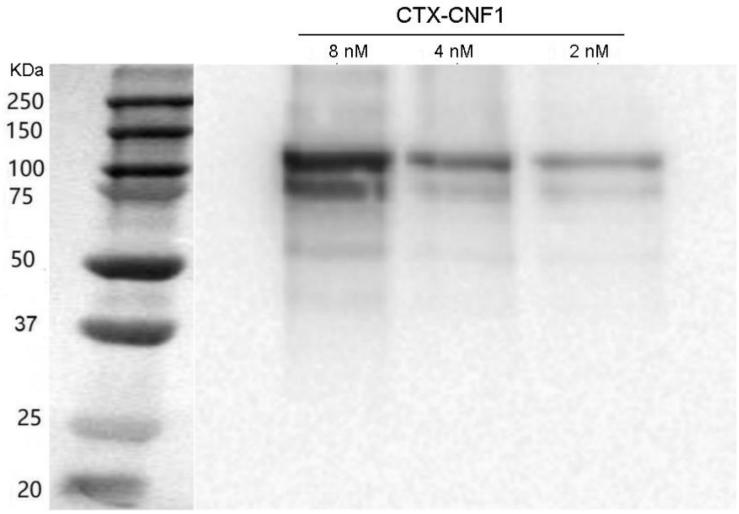

Figure S2. CNF1 antibody recognizes CNF1 catalytic domain. Immunoblot of CTX-CNF1, whose molecular weight is almost 118 KDa. This assay was done before proceeding with the tissues, in order to check the functionality of the antibody for CNF1. To address this issue, we tested it on the chimeric protein alone, diluted at different concentration (i.e. 8 nM, 4 nM, 2 nM). We found that the antibody for CNF1 catalytic domain was able to recognize our chimeric protein.
